# Assessment of Functional Status in Patients with Multiple Sclerosis Based on the Inflammatory Potential of Their Diet

**DOI:** 10.3390/nu17243951

**Published:** 2025-12-17

**Authors:** Sofía Fernández-Godino, Irene Cabrera-Martos, Alejandro Heredia-Ciuró, Araceli Ortiz-Rubio, María Granados-Santiago, Marie Carmen Valenza

**Affiliations:** 1 Department of Physical Therapy, Faculty of Health Sciences, University of Granada, 18016 Granada, Spain; sofiafg@correo.ugr.es (S.F.-G.); ahc@ugr.es (A.H.-C.); aortiz@ugr.es (A.O.-R.); cvalenza@ugr.es (M.C.V.); 2 Department of Nursing, Faculty of Health Sciences, University of Granada, 18016 Granada, Spain; mariagranados@ugr.es

**Keywords:** multiple sclerosis, diet, inflammation, functionality, dietary inflammatory index

## Abstract

Background: Multiple sclerosis (MS) is a chronic, inflammatory, and demyelinating disease of the central nervous system that predominantly affects young adults, particularly women, and is associated with progressive disability and a wide range of symptoms that impair functionality and quality of life. Recent research suggests that diet, especially its inflammatory potential, may influence the clinical course of the disease. We hypothesize that patients following a proinflammatory dietary pattern will exhibit poorer functional outcomes than those following an anti-inflammatory diet. Methods: An observational preliminary study was conducted, including 19 patients. Dietary inflammatory potential was assessed using the Dietary Inflammatory Index (DII), while functional status was measured with the Functional Assessment of MS (FAMS) scale. Results: Participants were divided into two groups according to their DII score: a group following a more pro-inflammatory diet (n = 10; 80% female; mean age 49.60 ± 10.63 years) and a group following a more anti-inflammatory diet (n = 9; 44.4% female; mean age 49.00 ± 10.79 years). The results show that patients with a proinflammatory dietary profile reported a higher symptom burden (FAMS symptoms score 20.70 ± 5.48 vs. 14.44 ± 7.05, *p* = 0.044), including greater fatigue as well as musculoskeletal and gastrointestinal complaints. In contrast, patients with an anti-inflammatory dietary profile reported fewer symptoms, greater energy and vitality, and higher intake of anti-inflammatory nutrients such as fiber, magnesium, and vitamin B6. No other significant between-group differences were observed. Conclusions: These findings suggest that dietary interventions aimed at reducing inflammation may improve functionality and quality of life in persons with MS. However, given the limited sample size, larger multicenter longitudinal studies are required to confirm these results. The findings of this study may provide preliminary evidence to inform future research.

## 1. Introduction

Multiple sclerosis (MS) is a chronic, inflammatory, demyelinating, and neurodegenerative disease of the central nervous system that predominantly affects young adults, particularly women [1,2]. MS is characterized by a wide spectrum of disabling symptoms that impair mobility, cognition, and overall quality of life [1]. Worldwide, MS affects more than 1.8 million people, with an increasing prevalence reported in several countries, including Spain [3]. Despite advances in pharmacological therapies aimed at modulating the immune response, no definitive cure exists, and disease progression continues to compromise patient functionality.

The etiology of MS is multifactorial and involves a complex interaction between genetics and environmental influence. It has been shown that factors such as Epstein–Barr virus, vitamin D deficiency, smoking, obesity, and immune dysregulation have been implicated in disease onset [4]. In addition, previous evidence suggests that MS disability is associated with modifiable lifestyle factors, including dietary habits, smoking, physical activity, alcohol consumption, and plant-based omega-3 supplementation [5]. Accordingly, increasing attention has been directed toward lifestyle factors, particularly diet, as potentially modifiable contributors to disease progression and symptom burden [6,7]. Evidence suggests that dietary patterns may influence systemic and neuroinflammation through their pro- or anti-inflammatory properties [8]. Proinflammatory diets, characterized by high intake of saturated fats, sugars, and processed foods, have been associated with worsening inflammation and neurodegeneration in patients with MS. In contrast, anti-inflammatory diets rich in fruits, vegetables, fiber, and omega-3 fatty acids may confer protective effects and improve clinical outcomes [9]. The nutrients have been shown to influence MS pathology through oxidative injury and gut–brain axis modulation [10]. In this sense, oxidative stress enhances inflammation and causes myelin injury, provoking cell death. Also, those diets rich in saturated fats and processed foods exacerbate neuroinflammation, promote Th1/Th17 polarization, and disease progression. In contrast, diets rich in monounsaturated and omega-3 fatty acids, dietary fiber, and balanced tryptophan metabolism are linked to anti-inflammatory immune regulation and increased regulatory T-cell activity. Additionally, fiber-derived short-chain fatty acids and omega-3 metabolites support gut barrier integrity [11]. While growing evidence suggests that diet may influence inflammatory activity in MS, few studies have specifically examined the impact of dietary inflammatory potential on functional performance [6]. Given that functional impairment is a major determinant of long-term disability and quality of life in MS, clarifying this association is clinically relevant.

This preliminary study aims to explore the functional performance of patients with MS in relation to the inflammatory potential of their habitual diet, as measured by the Dietary Inflammatory Index (DII). We hypothesize that patients following a proinflammatory dietary pattern will exhibit poorer functional outcomes than those following an anti-inflammatory diet. The findings of this study may provide preliminary evidence to inform future research and the design of targeted nutritional interventions designed to enhance functionality and quality of life in this population.

## 2. Materials and Methods

### 2.1. Design and Ethical Considerations

This observational preliminary study was conducted at the facilities of the Granadian MS Association (Asociación Granadina de Esclerosis Múltiple, AGDEM) in Armilla, Granada, Spain. All participants provided written informed consent after receiving detailed information about the study objectives, procedures, potential benefits, and data management. The study was approved by the Provincial Ethics Committee of Granada on 11 April 2025, and adhered to the ethical principles outlined in the Declaration of Helsinki [12].

### 2.2. Population and Participant Selection Procedure

Participants were individuals diagnosed with MS who were members of AGDEM. Convenience sampling was employed. The inclusion criteria required participants to be over 18 years old, diagnosed with MS, and to possess sufficient cognitive and communicative abilities to complete the assessments related to diet and functional performance. Exclusion criteria included the presence of communication impairments, conditions interfering with the assessment process, severe cognitive deterioration, or a diagnosis of concomitant neurological disorders.

### 2.3. Data Collection and Variables

Data collection was carried out in May and June 2025. Each participant was thoroughly informed about the study objectives, assured the confidentiality of their data, and reminded of their right to withdraw at any time. Written informed consent was obtained before initiating the assessment procedures. A single investigator administered all assessments to maintain consistency.

At the beginning of the evaluation, a structured interview was conducted to collect sociodemographic and clinical data. The variables collected included sex, age, body mass index, MS phenotype, disease duration since diagnosis, marital status, educational level, employment status, and living arrangements. In addition, gait capacity was assessed using the MS Walking Scale—12 items (MSWS-12%), a validated questionnaire designed specifically for persons with MS [13,14].

The primary variables analyzed were the inflammatory nutritional profile and functional status. The dietary profile was assessed using the DII [15]. This is a tool used to measure the inflammatory potential of an individual’s habitual diet by analyzing specific dietary components with documented effects on systemic inflammation markers. It generates a score on a scale where positive numbers indicate foods that tend to increase inflammation, and negative numbers indicate foods that help to reduce it. The index works by giving each food or nutrient a score based on whether it has been shown to raise or lower inflammation, allowing for an overall assessment of the diet’s inflammatory effect [15]. The development of this index is based on a systematic review of literature examining associations between foods, nutrients, and bioactive compounds with six inflammatory biomarkers: interleukin-1β (IL-1β), interleukin-4 (IL-4), interleukin-6 (IL-6), interleukin-10 (IL-10), tumor necrosis factor-alpha (TNF-α), and C-reactive protein (CRP).

Dietary intake was assessed using a 3-day food diary (two weekdays and one weekend day) to register habitual dietary intake. Household measures and portion-size photographs were used to enhance accuracy. In cases of uncertainty, participants received clarifications from the evaluator to resolve any doubts regarding the dietary record. Nutrient quantities and nutritional components required for DII calculation were derived from these records and adjusted to individual intake based on the meals and portion sizes reported by participants in the three-day 24 h dietary record.

The DII score was calculated based on the relative intake of up to 45 dietary components. Each component was assigned to an inflammatory coefficient based on its pro- or anti-inflammatory effects. Intake values were standardized using an international database, converted into z-scores, transformed into centered percentiles, and multiplied by their respective coefficients. The sum of these products yielded the individual total DII score [15].

To estimate the nutritional composition of consumed foods, two databases were used: the Spanish Food Composition Database (BEDCA) was used as the primary source, and the Italian Research Centre for Food and Nutrition (CREA) was consulted when specific items or nutrients were not available in BEDCA. This database was selected for its alignment with Mediterranean and Spanish dietary patterns. For analysis purposes, participants were distributed into two groups: a proinflammatory or an anti-inflammatory group to facilitate comparison of functional and symptomatic outcomes across dietary patterns [16,17].

Functional status was assessed using the Functional Assessment of MS (FAMS) tool [18], which includes 58 items across the following domains: mobility, symptoms, emotional well-being, general satisfaction, cognition and fatigue, and social and family well-being. Items were rated on a 5-point scale (0 = none; 4 = very much). Subscale scores range from 0 to 28 points, except for the cognition and fatigue subscale, which ranges from 0 to 36 points. Item scores were adjusted according to the correction template. The total FAMS score was calculated by summing all subscale scores, with higher scores indicating better quality of life. It was validated in Spain [19].

The total duration of the in-person evaluation was limited to a maximum of 45–50 min per patient to minimize fatigue and avoid interference with results.

### 2.4. Data Analysis Procedure

Data were analyzed using the Statistical Package for the Social Sciences (IBM Corp., Released 2013. IBM SPSS Statistics for Windows, Version 22.0. Armonk, NY, USA: IBM Corp). Participants were symmetrically distributed into two groups based on their DII scores. A descriptive analysis was performed for all collected variables, calculating means and standard deviations for quantitative variables and frequencies for qualitative variables.

The normality assumption was tested using the Shapiro–Wilk test. Differences between groups were analyzed using Pearson’s chi-square test for qualitative variables and Student’s *t*-test for quantitative variables. When the normality assumption was not met, the non-parametric Mann–Whitney test was applied. A *p*-value < 0.05 was considered statistically significant for all tests. DII scores could not be obtained for two participants due to missing information; in these cases, missing data were handled by mean imputation

## 3. Results

A total of 19 patients (n = 19) from AGDEM participated in the study. The proinflammatory diet group included 10 patients, while the anti-inflammatory diet group included 9 patients.

The descriptive characteristics of the patients are presented in Table 1.

Both groups presented mostly comparable sociodemographic and clinical profiles. No statistically significant differences were found between the descriptive variables of the two groups.

In the proinflammatory diet group, there were 8 women and 2 men, whereas the anti-inflammatory diet group included 4 women and 5 men. The mean age was similar in both groups. Although the proinflammatory group had a predominance of females and a higher proportion of patients with relapsing-remitting MS, these differences were not statistically significant. The mean time since diagnosis was slightly higher in the proinflammatory group, but did not reach significance.

The distributions of marital status, educational level, and employment status were balanced between both groups, with retirement and cohabitation being predominant, factors that may influence social support and quality of life. The mean MSWS-12 score was also similar in both groups. Overall, the homogeneity in most variables allows greater confidence in attributing observed functional and symptomatic differences to dietary patterns rather than to potential confounding factors.

Table 2 shows the characteristics of the DII according to diet type in the patients included in the study. Significant between-group differences were found for vitamin B6 (mg) (*p* = 0.016); the anti-inflammatory group consumed significantly more vitamin B6, a nutrient with anti-inflammatory and antioxidant properties. The intake of β-carotene (μg), an antioxidant and precursor to vitamin A, also showed significant between-group differences (*p* = 0.037) and was considerably higher in the anti-inflammatory group (4682 ± 4114.37 µg). The anti-inflammatory group also exhibited a significantly higher intake of eugenol, a compound found in spices such as clove, with recognized anti-inflammatory effects. Fiber consumption was higher in the anti-inflammatory group (*p* = 0.040). Magnesium intake was significantly higher in the anti-inflammatory group. Niacin (B3) intake was notably higher, with a mean of 24.73 and a standard deviation of 12.11 in the anti-inflammatory group. Omega-3 fatty acid consumption, known for its anti-inflammatory effects, was much higher in the anti-inflammatory group. Flavanones, antioxidant compounds found in citrus fruits, were significantly higher in the anti-inflammatory group, with a mean of 3.85 and a standard deviation of 4.61.

No statistically significant differences were observed for the other variables between the groups. Overall, the anti-inflammatory diet group consumed significantly more nutrients and bioactive compounds with potential anti-inflammatory and antioxidant effects (vitamin B6, β-carotene, eugenol, fiber, magnesium, niacin, omega-3 fatty acids, and flavanones).

The functional characteristics of the study patients according to dietary profile are presented in Table 3.

The table displays the functional profiles of MS patients based on their diet, distinguishing between a proinflammatory diet group (n = 10) and an anti-inflammatory diet group (n = 9). In most dimensions assessed by the FAMS scale, no statistically significant differences were observed between groups, with similar mean values in overall functionality.

However, a significant difference was noted in the FAMS Symptoms subscale, where the proinflammatory diet group showed a higher mean score (20.70 ± 5.48) compared to the anti-inflammatory diet group (14.44 ± 7.05), with a *p*-value of 0.044. This indicates that patients with an anti-inflammatory diet reported a lower symptomatic burden. These results suggest that although overall functionality is comparable between groups, an anti-inflammatory diet may be associated with better perception and control of specific disease symptoms.

## 4. Discussion

This study aimed to assess functional performance in patients with MS according to the inflammatory potential of their diet. The results demonstrate significant differences in the Symptoms subscale of the FAMS tool between patients following a more proinflammatory diet and those adopting an anti-inflammatory diet. Specifically, participants with a less proinflammatory diet reported lower levels of fatigue, musculoskeletal pain, digestive symptoms such as nausea, and better perceptions of energy and vitality compared to the group with a more proinflammatory diet.

Currently, there is no direct evidence linking nutritional status with the onset of MS. However, lifestyle changes, such as modifications in dietary habits, have been observed to improve disease progression by acting on metabolism and the microbiota, which are responsible for immune system functioning [8]. The relationship between diet and clinical progression of MS is an area of growing interest, especially regarding patient functionality. Recent scientific literature supports the hypothesis that dietary patterns can modulate both disease progression and quality of life, as well as the risk of associated complications [20].

The findings of this study align with recent scientific literature. Several previous studies have shown that dietary patterns including high consumption of ultra-processed foods, saturated fats, and sugars promote systemic inflammation and neurodegeneration, contributing to worse clinical outcomes in MS. Saturated fats activate proinflammatory pathways affecting innate immunity and increasing relapse risk [20]. These dietary habits are associated with greater disability and faster functional decline. Conversely, diets rich in oily fish, fruits, vegetables, and fiber, with abundant flavonoids and polyphenols, have been associated with lower risk of demyelination, reduced fatigue, and decreased disability, promoting a more favorable course of MS. In line with this study, our results show that the group with an anti-inflammatory dietary pattern had significantly higher fiber intake, reinforcing its relevant role in modulating inflammation and its potential positive impact on disease progression. The systematic review conducted by Harirchian et al. [21] analyzed the impact of various diets in MS patients, evaluating outcomes such as fatigue, quality of life, disability, and relapse rates. The authors found that vegetarian, low-fat, low-calorie, and low-protein diets showed moderate positive effects on fatigue and disability symptoms. Additionally, supplementation with vegetable oils and consumption of flavonoid-rich foods demonstrated further benefits. These data support our findings, as the anti-inflammatory diet group had markedly higher flavanone intake. However, the authors caution about risks associated with highly restrictive diets and emphasize the importance of maintaining adequate nutritional balance.

Khattab et al. [22] conducted a review highlighting that higher diet quality is associated with lower disability levels and less severe symptoms in MS. The work underscores the importance of macronutrients, vitamins, and minerals, noting that adequate intake may be key to slowing disease progression and optimizing quality of life. In line with this, the present study found that the anti-inflammatory diet group had significantly higher magnesium and vitamin B6 intake compared to the proinflammatory group. These nutrient levels may contribute to better modulation of inflammatory responses, neuromuscular function, and protection against oxidative stress, key factors in MS clinical outcomes. Thus, empirical results support the hypothesis that a higher-quality diet, rich in essential micronutrients, is associated with less severe symptoms and improved functionality, as noted by Khattab et al. [22]. Current evidence shows no significant effect of gluten-free diets on MS [23].

Recent research has also linked proinflammatory diets with the onset and exacerbation of symptoms such as depression and anxiety, particularly long-term [24]. Inflammation alters neurotransmission of substances such as serotonin and dopamine, which play fundamental roles in emotional regulation [9]. This study did not find significant results in this regard, as specific evaluation of symptoms and socioemotional aspects was not conducted, limiting the ability to establish associations in this area. Data obtained suggest that adopting a more anti-inflammatory diet could be an effective tool to reduce symptom burden, thereby improving quality of life and disease progression. Previous evidence suggests that there is an interaction between dietary interventions and pharmacological therapy in MS. For example, corticosteroid use can impair calcium absorption and contribute to osteoporosis. In addition, symptoms frequently reported by people with MS, such as anorexia, nausea, diarrhea, and vomiting, may adversely affect nutritional intake [25]. However, evidence addressing whether dietary interventions can reduce pharmacological side effects remains limited. Future studies are needed to specifically address the interaction between pharmacological treatments, such as corticosteroids, diet, and the microbiome in patients with MS. Overall, current evidence supports that healthy and balanced nutritional patterns may play a protective role against MS progression, disability, and functional decline. In particular, adherence to a Mediterranean diet has been associated with reduced inflammation and improvements in symptoms, including reduced fatigue and body mass index, as well as improved quality of life [26,27]. These results support the establishment of specific, personalized dietary recommendations for the MS population, favoring an integrative approach.

This study presents several limitations. First, the cross-sectional design and the potential bias associated with dietary intake assessment using a 3-day food diary are important limitations of this study. Future studies should incorporate longer intervention periods and laboratory analyses to validate and extend our findings. In addition, although the database CREA was used only when specific nutrients were not available in BEDCA, using two databases from different countries may have introduced some bias. The small sample size limits the generalizability of results to the broader MS population. However, the present study was designed as a preliminary study, and the results should be interpreted as hypothesis-generating rather than confirmatory. Although collaboration with the AGDEM association facilitated the dissemination of the study, the pool of potential participants was inherently constrained by the limited number of patients who attend the association. Additionally, all participants belonged to the same MS association, potentially limiting sample representativeness and reducing variability in patient profiles.

Integrating dietary intervention into comprehensive MS management may improve functionality and quality of life. It is essential to advance evidence-based clinical guidelines and promote research on personalized dietary recommendations tailored to disease phenotype, age, nutritional status, and comorbidities. The diet emerges as a highly relevant modifiable factor in MS progression. Promoting healthy dietary patterns, combined with individualized nutritional counseling, should be considered a key strategy in preventing and managing disability in this population.

## 5. Conclusions

This study provides evidence on the influence of the inflammatory potential of diet on symptomatology in individuals with MS. The results reveal significant differences in the Symptoms subscale of the FAMS questionnaire between patients following a proinflammatory diet and those adopting an anti-inflammatory diet.

Specifically, the proinflammatory diet group exhibited a higher symptom burden, characterized by increased levels of fatigue, musculoskeletal pain, digestive symptoms such as nausea, and poorer perceptions of energy and vitality. In contrast, patients with an anti-inflammatory diet reported lower intensity and frequency of these symptoms, suggesting a protective effect of this dietary pattern on quality of life and functional well-being. These findings support the hypothesis that diet, particularly its inflammatory profile, may modulate not only the clinical progression of MS but also the daily experience of symptoms that most affect patients’ autonomy and functionality.

The anti-inflammatory diet group showed a higher intake of fiber, magnesium, and vitamin B6, as estimated from the DII; these variables are commonly related to systemic inflammation and oxidative stress in the context of the disease. However, further multicenter studies with larger sample sizes and longitudinal follow-up are needed to confirm and expand these results and to establish specific, personalized dietary recommendations for the MS population.

## Figures and Tables

**Table 1:** Descriptive characteristics of the patients included in the study.

Variables	Patients with a Pro-Inflammatory Diet (n = 10)	Patients with an Anti-Inflammatory Diet (n = 9)	*p* Value
Gender n (% female)	8 (80)	4 (44,4)	0.109
Age (years)	49.60 ± 10.63	49.00 ± 10.79	0.904
BMI (kg/m^2^) (mean ± SD)	24.07 ± 2.44	24.83 ± 3.42	0.580
Type of EM n (%)			0.119
Relapsing-remitting	90 (9)	5 (55,6)	
Primary progressive	10 (1)	4 (44.4)	
Duration since diagnosis (years)	11.70 ± 9.86	8 ± 5.33	0.424
Marital status n (%)			0.869
Single	5 (50)	5 (55.6)	
Married	3 (30)	3 (33.3)	
Divorced	2 (20)	1 (11.1)	
Educational level	1 (10)	0 (0)	0.178
Secondary School Graduate	1 (10)	4 (44.4)	
Vocational training	8 (80)	5 (55.6)	
University studies			
Employment status			0.399
Full-time work	2 (20)	2 (22.2)	
Unemployed	2 (20)	0 (0)	
Retired	6 (60)	6 (66.7)	
Other situation	0 (0)	1 (11.1)	
Living with another person	7 (70)	5 (55.6)	0.430
MSWS12%	63.50 ± 19.83	59.81 ± 27.21	0.738

SD: Standard deviation; BMI: Body mass index; MSWS-12%: Twelve-item Multiple Sclerosis Walking Scale.

**Table 2:** Dietary Inflammatory Index characteristics according to diet type in the patients included in the study.

Variables (Mean ± SD)	Patients on a Pro-Inflammatory Diet (n = 10)	Patients with an Anti-Inflammatory Diet (n = 9)	*p* Value
Alcohol (g)	1.54 ± 3.19	3.47 ± 3.80	0.246
Vitamin B12 (µg)	4.19 ± 1.56	5.58 ± 2.44	0.156
Vitamin B6 (mg)	1.27 ± 0.29	2.46 ± 1.40	0.018
β-carotene (µg)	1569.25 ± 1388.67	4682.75 ± 4114.37	0.037
Caffeine (mg)	51.66 ± 30.77	50.54 ± 25.02	0.932
Carbohydrates (g)	820.54 ± 2101.05	279.98 ± 190.92	0.454
Cholesterol (mg)	256.85 ± 103.90	355.50 ± 205.35	0.197
Energy (kcal)	1456.82 ± 219.27	1913.51 ± 948.12	0.156
Eugenol (mg)	0.166 ± 0.52	1.66 ± 1.72	0.017
Total fat (g)	63.37 ± 19.09	80.72 ± 35.83	0.199
Fiber (g)	15.64 ± 4.76	31.98 ± 22.78	0.040
Folic acid (µg)	257.11 ± 78.12	354.03 ± 195.04	0.165
Garlic (g)	0.50 ± 0.67	0.79 ± 0.91	0.443
Ginger (g)	0.39 ± 1.09	4.07 ± 9.55	0.241
Magnesium (mg)	213.50 ± 50.96	284.88 ± 91.91	0.049
Fe (mg)	8.25 ± 2.12	24.50 ± 34.61	0.156
MUFA (g)	34.93 ± 14.47	39.65 ± 16.81	0.519
Niacin (B3) (mg)	14.13 ± 4.50	24.73 ± 12.11	0.019
n-3 fatty acids (g)	2.14 ± 1.30	3.48 ± 2.40	0.142
n-6 fatty acids (g)	8.11 ± 4.66	12.44 ± 3.53	0.037
Onion (g)	11.16 ± 20.80	37.58 ± 39.25	0.080
Protein (g)	68.77 ± 12.86	90.79 ± 49.15	0.189
PUFA (g)	13.73 ± 6.84	12.02 ± 3.76	0.517
Riboflavin (B2) (mg)	1.80 ± 1.22	2.71 ± 1.32	0.136
Saffron (g)	0.63 ± 1.82	0.79 ± 1.24	0.832
Saturated fat (g)	44.03 ± 58.09	50.45 ± 36.80	0.780
Se (µg)	60.65 ± 33.39	97.03 ± 64.46	0.135
Thiamin (B1) (mg)	1.10 ± 0.69	1.87 ± 1.56	0.172
Trans fat (g)	1.50 ± 4.38	41.50 ± 93.22	0.192
Turmeric (mg)	46.85 ± 104.67	199.97 ± 265.10	0.109
Vitamin A (RE µg)	798.77 ± 1103.10	977.43 ± 412.50	0.654
Vitamin C (mg)	127.01 ± 217.19	148.59 ± 79.16	0.782
Vitamin D (µg)	10.45 ± 24.59	16.45 ± 20.95	0.577
Vitamin E (mg)	10.85 ± 9.08	13.93 ± 4.42	0.369
Zn (mg)	7.88 ± 2.65	13.42 ± 11.83	0.167
Green/black tea (g)	0.58 ± 0.96	0.75 ± 0.89	0.683
Flavan-3-ol (mg)	25.20 ± 28.73	46.41 ± 44.08	0.226
Flavones (mg)	0.68 ± 0.85	2.97 ± 3.44	0.057
Flavonols (mg)	2.36 ± 3.08	10.72 ± 15.80	0.119
Flavonones (mg)	0.59 ± 0.77	3.85 ± 4.61	0.041
Anthocyanidins (mg)	5.46 ± 7.94	4.64 ± 7.52	0.821
Isoflavones (mg)	0.35 ± 1.05	0.17 ± 0.23	0.620
Pepper (g)	0.14 ± 0.42	0.24 ± 0.31	0.568
Thyme/orégano (mg)	0.07 ± 0.21	0.26 ± 0.55	0.314
Rosemary (mg)	0.03 ± 0.10	0.13 ± 0.24	0.249

MUFA: monounsaturated fatty acids; PUFA: polyunsaturated fatty acids.

**Table 3:** Functional characteristics of the study patients according to diet type.

Variables (Mean ± SD)	Patients on a Pro-Inflammatory Diet (n = 10)	Patients with an Anti-Inflammatory Diet (n = 9)	*p* Value
FAMS Mobility	13.70 ± 4.11	12.67 ± 7.31	0.705
FAMS symptoms	20.70 ± 5.48	14.44 ± 7.05	0.044
FAMS emotional well-being	17.80 ± 7.81	16.89 ± 7.36	0.797
FAMS overall satisfaction	17.60 ± 6.06	17.00 ± 5.92	0.830
FAMS fatigue	15.30 ± 8.31	13.78 ± 8.70	0.701
FAMS family/social well-being	16.90 ± 6.57	17.89 ± 7.56	0.764
FAMS TOTAL	102.00 ± 21.07	92.67 ± 33.79	0.475

SD: Standard deviation; FAMS: Functional Assessment of Multiple Sclerosis.

## Data Availability

The data presented in this study are available on request from the corresponding author due to ethical reasons.

## Abbreviations

The following abbreviations are used in this manuscript:

DII	Dietary Inflammatory Index
FAMS	Functional Assessment of Multiple Sclerosis
MS	Multiple sclerosis
MSWS-12%	Twelve items-Multiple Sclerosis Walking Scale
MUFA	Monounsaturated fatty acids
PUFA	Polyunsaturated fatty acids
